# The Diagnostic Value of Immunohistochemistry and Biomarkers in the Diagnosis of Mesothelioma in Pleural/Peritoneal Effusions

**DOI:** 10.1002/dc.70147

**Published:** 2026-05-20

**Authors:** Suzanne M. Selvaggi

**Affiliations:** ^1^ Department of Pathology and Laboratory Medicine University of Wisconsin School of Medicine and Public Health Madison Wisconsin USA

**Keywords:** BAP1, biomarkers, mesothelioma, MTAP, pleural/peritoneal effusions, specialized techniques

## Abstract

**Background:**

Mesothelioma is a rare tumor that arises from the serosal cells lining the serous cavities, most commonly the pleura followed by the peritoneum. Presenting symptoms include pleuritic pain and bloody effusions that recur. Although cytologic analysis cannot determine the mesothelial subtype or in situ from invasive disease, the application of biomarkers to serous effusions has aided in distinguishing benign from malignant mesothelial cells. This study evaluates the diagnostic value of specialized techniques in the cytologic diagnosis of mesothelioma pre‐ and post‐implementation of biomarkers and a robust immunohistochemical (IHC) panel.

**Methods:**

From January 1, 2013, through December 31, 2025, 26 effusions (20 pleural, six peritoneal) from 16 males and four females (mean, 72 years; median, 75 years) were evaluated and contained atypical mesothelial cells. In each case, ThinPrep slides were made for cytologic analysis and cell blocks were prepared for IHC and biomarker evaluation. Prior to 2017, the IHC markers were limited, whereas after that time, biomarkers including BAP1 and MTAP were available.

**Results:**

Cytologically the effusions were cellular and contained atypical, enlarged mesothelial cells arranged singly and in clusters with a normal nuclear/cytoplasmic ratio. Five effusions (2013–2015) were diagnosed as suspicious for mesothelioma based on the cytologic findings and a limited IHC panel (calretinin, CK 5/6, TTF‐1). The diagnosis was confirmed on biopsy/resection specimens. For 17 effusions (2017–2025), an expanded IHC and biomarker panel aided in the cytologic diagnosis of mesothelioma. Three effusions were from patients with a known history of pulmonary mesothelioma, so IHC stains were not performed. Histologic confirmation was done in 23 cases.

**Conclusions:**

The application of IHC and biomarkers in combination with cytomorphology aids in the diagnosis of mesothelioma on fluid cytology.

## 1 Introduction

The embryonic coelomic cavity gives rise to the serous cavities: pleural, peritoneal, and pericardial. Each cavity is lined by a thin layer of mesothelial cells and each contains a small amount of fluid. Accumulation of fluid is referred to as an effusion. Normal cellular elements found in serous fluids include erythrocytes, scattered neutrophils, and mesothelial cells which vary in number from scanty to numerous. Mesothelioma is a tumor that arises from the surface serosal cells lining the serous cavities with the pleura the most common. A strong link between asbestos exposure and the development of mesothelioma was first reported in 1960 [1]. The majority of patients present with pleuritic pain associated with recurrent, bloody pleural effusions which usually contain malignant mesothelial cells [2].

Mesothelial cells have many faces in effusions ranging from reactive to hyperplastic. They can be mononucleate, binucleate, and/or multinucleated. Cytoplasmic vacuolization may be present, varying from tiny vacuoles to large vacuoles that displace the nucleus. A tip‐off to their origin is the “windows” that form between the cells.

Several cytologic features are taken into consideration when considering a diagnosis of mesothelioma. Are the cells in question mesothelial, and if so, are they neoplastic. Does the cellularity, cellular arrangement and morphologic features favor a diagnosis of mesothelioma. Interestingly, the morphologic features of benign mesothelial cells overlap those of neoplastic mesothelial cells, both in presentation and cellular morphology [3]. The most significant features pointing to the diagnosis of malignant mesothelioma are the large number and increased size of mesothelial cells singly and in clusters, multinucleation and the prominence of the nucleoli [4]. Although the morphologic diagnosis of mesothelioma is challenging, given the overlap with benign, reactive hyperplastic mesothelial cells, the use of fluorescence in situ hybridization, immunohistochemistry, and biomarkers can aid in distinguishing the two.

A definitive diagnosis of mesothelioma is usually reached with a tissue biopsy or on surgical resection specimens. However, a pleural/peritoneal effusion is often the first sign of malignancy. A cytologic examination of effusion fluid is far less invasive and can be readily performed, particularly in patients who cannot tolerate invasive procedures. At early stages of the disease, a definitive cytologic diagnosis can expedite staging of the patient and formulate a management plan.

This study reports on the value of immunohistochemistry and biomarkers in the diagnosis of mesothelioma in pleural/peritoneal effusions.

## 2 Materials and Methods

### 2.1 Patients

From January 1, 2013 through December 31, 2025, the Cytopathology Laboratory of the University of Wisconsin Hospital processed and analyzed 26 serous fluids (20 pleural; 12 left and eight right, and six peritoneal). Each showed the presence of atypical mesothelial cells. The patients, 16 males and four females, ranged in age from 52 to 92 years (mean, 72 years; median, 75 years). Presenting symptoms included chest pain and/or abdominal pain and dyspnea.

Five patients had two serous fluids collected; two with pleural and peritoneal effusions and three with pleural effusions. Three patients had a history of mesothelioma of the lung/peritoneum (1–2 years prior) at the time of effusion sampling. Histologic follow‐up was available in 23 cases; 15 pleural biopsies, five peritoneal biopsies, one pleural biopsy and follow‐up pneumonectomy, one peritoneal biopsy and omental resection, and one right thoracotomy, decortication, and pleurectomy. One pleural fluid was seen in consultation without histologic follow‐up, one patient sought care at another institution, and one patient, due to advanced age, was managed with pleural drainage, only.

### 2.2 Specimen Preparation and Processing

Pleural and peritoneal fluid specimens are submitted fresh (50 mL) to the Cytopathology laboratory and processed according to the laboratory's procedures. Briefly, 40 mL of fluid is transferred to a labeled centrifuge tube, vortexed and centrifuged at 1712 rpm for 10 min and the supernatant is removed and discarded. The cellular pellet is reconstituted with 40 mL of Cytolyt Solution (Hologic Inc., Marlborough, MA, USA.), the tube is vortexed and centrifuged. The supernatant is discarded and the cellular material is transferred to the ThinPrep PreservCyt Solution (Hologic Inc., Marlborough, MA, USA) for processing. Thin‐layer slides were prepared using the ThinPrep 5000 Automated Slice Processor (Hologic Inc., Marlborough, MA, USA) according to the manufacturer's instructions. The slides are manually removed from the processor and stained by the Papanicolaou method. The remaining material was processed for cell block preparation utilizing the plasma‐thrombin clot technique [5] and paraffin embedded. Cell blocks were included in the validation process for the IHC/biomarkers utilized to ensure diagnostic accuracy.

Hematoxylin–eosin and immunohistochemical stains were performed on the cell block material using the avidin‐biotin peroxidase complex method on the automated NEXES system (Ventana Medical Systems, Tucson, AZ, USA). The following prediluted antibodies were used: pan‐keratin (AE1/AE3, Zymed Laboratories, South San Francisco, CA, USA). Cytokeratin 5/6 (Ventana Medical Systems, Tucson, AZ, USA), calretinin (Ventana Medical Systems, Tucson, AZ, USA), WT‐1 (Ventana Medical Systems, Tucson, AZ, USA), claudin 4 (BioCare Medical, Pacheco, CA, USA), TTF‐1/SPT24 (BioCare Medical, Pacheco, CA, USA), and BAP1 (BioSB Inc., Goleta, CA, USA). MTAP immunohistochemical staining was performed at an outside reference lab and interpreted by our pathologists.

## 3 Results

From 2013 through 2015, five effusions from three patients, three pleural and two peritoneal, contained atypical mesothelial cells with cytologic feature suspicious for mesothelioma. The immunohistochemical panel was limited and showed strong staining positivity for calretinin and CK 5/6 and was negative for TTF‐1. Subsequent pleura and peritoneal biopsies, pleurectomy and peritoneal resection confirmed the diagnosis of mesothelioma (Table 1).

**TABLE 1:** Cytologic, immunohistochemical, and histologic diagnosis.

	Number of cases	Cytologic diagnosis	IHC results	Histologic diagnoses
2013–2015 (years)				
Pleural effusions	3	Suspicious for mesothelioma	Calretinin(+), CK5/6(+), TTF‐1(−)	Epithelioid mesothelioma
Peritoneal effusions	2	Suspicious for mesothelioma	Calretinin(+), CK5/6(+), TTF‐1(−)	Epithelioid mesothelioma
2017–2025 (years)				
Pleural effusions	7	Mesothelioma	Calretinin(+), CK5/6(+), WT‐1(+), BAP1(−), MTAP(−), claudin 4(−), TTF‐1(−)	Epithelioid mesothelioma
Peritoneal effusions	3	Mesothelioma	Calretinin(+), CK5/6(+), WT‐1(+), BAP1(−), MTAP(−), claudin 4(−), TTF‐1(−)	Epithelioid mesothelioma
Pleural effusions	3	Mesothelioma	Calretinin(+), CK5/6(+), WT‐1(+), BAP1(−), claudin 4(−), TTF‐1(−)	Epithelioid mesothelioma
Peritoneal effusions	1	Mesothelioma	Calretinin(+), CK5/6(+), WT‐1(+), BAP1(−), claudin 4(−), TTF‐1(−)	Epithelioid mesothelioma
Pleural effusions	1	Mesothelioma	BAP1(−)	Epithelioid mesothelioma
Pleural effusions	3	Mesothelioma	Calretinin(+), CK5/6(+), WT‐1(+), BAP1(−), claudin 4(−), TTF‐1(−)	No biopsy
Pleural effusions	3	Mesothelioma	NA	Mesothelioma

From 2017 through 2025, 21 effusions from 17 patients, 17 pleural and four peritoneal, contained atypical mesothelial cells. A robust immunohistochemical panel consisting of CK 5/6, calretinin, WT‐1, BAP1, claudin 4, and TTF‐1 supported a diagnosis of mesothelioma in 14 cases, of which MTAP was also performed in 10 cases. One pleural effusion had only BAP1 staining and three pleural effusions, one pleural and one peritoneal, did not have immunohistochemical staining, as the patients had a prior history of pulmonary mesothelioma (Table 1).

**FIGURE 1:**
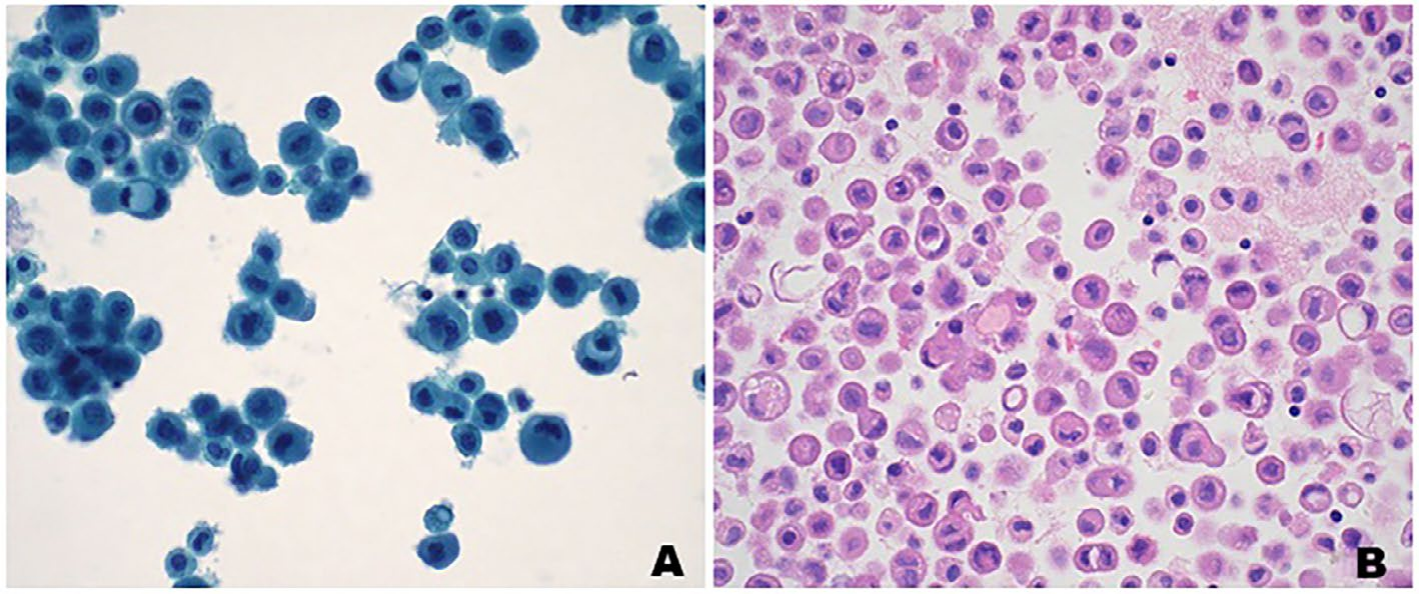
(A) Enlarged atypical mesothelial cells arranged singly. The nuclear/cytoplasmic ratio is unremarkable, and cell clasping is present (ThinPrep, Papanicolaou stain, 60×). (B) Cell clasping and cannibalism are present (cellblock, H&E stain, 60×). [Color figure can be viewed at wileyonlinelibrary.com]

**FIGURE 2:**
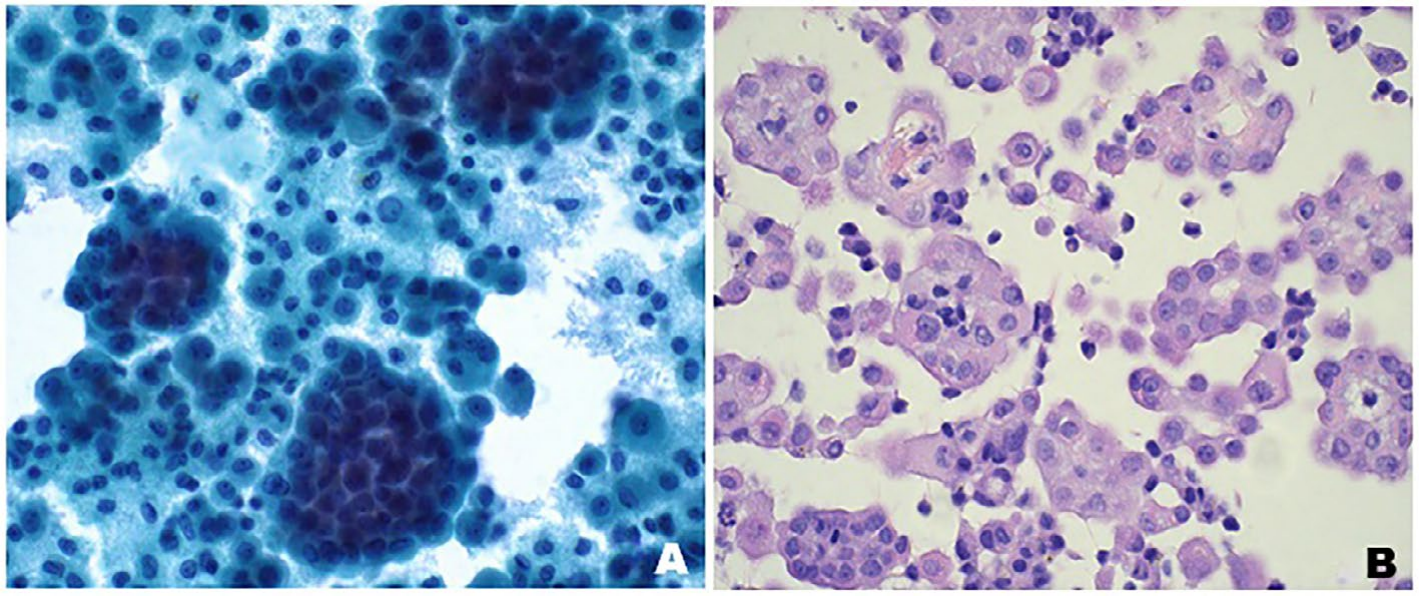
(A) Atypical mesothelial cells arranged singly and in groups (ThinPrep, Papanicolaou stain, 60×). (B) Hollow rings lined by atypical mesothelial cells containing centrally located nuclei with prominent nucleoli (cell block, H&E stain, 60×). [Color figure can be viewed at wileyonlinelibrary.com]

**FIGURE 3:**
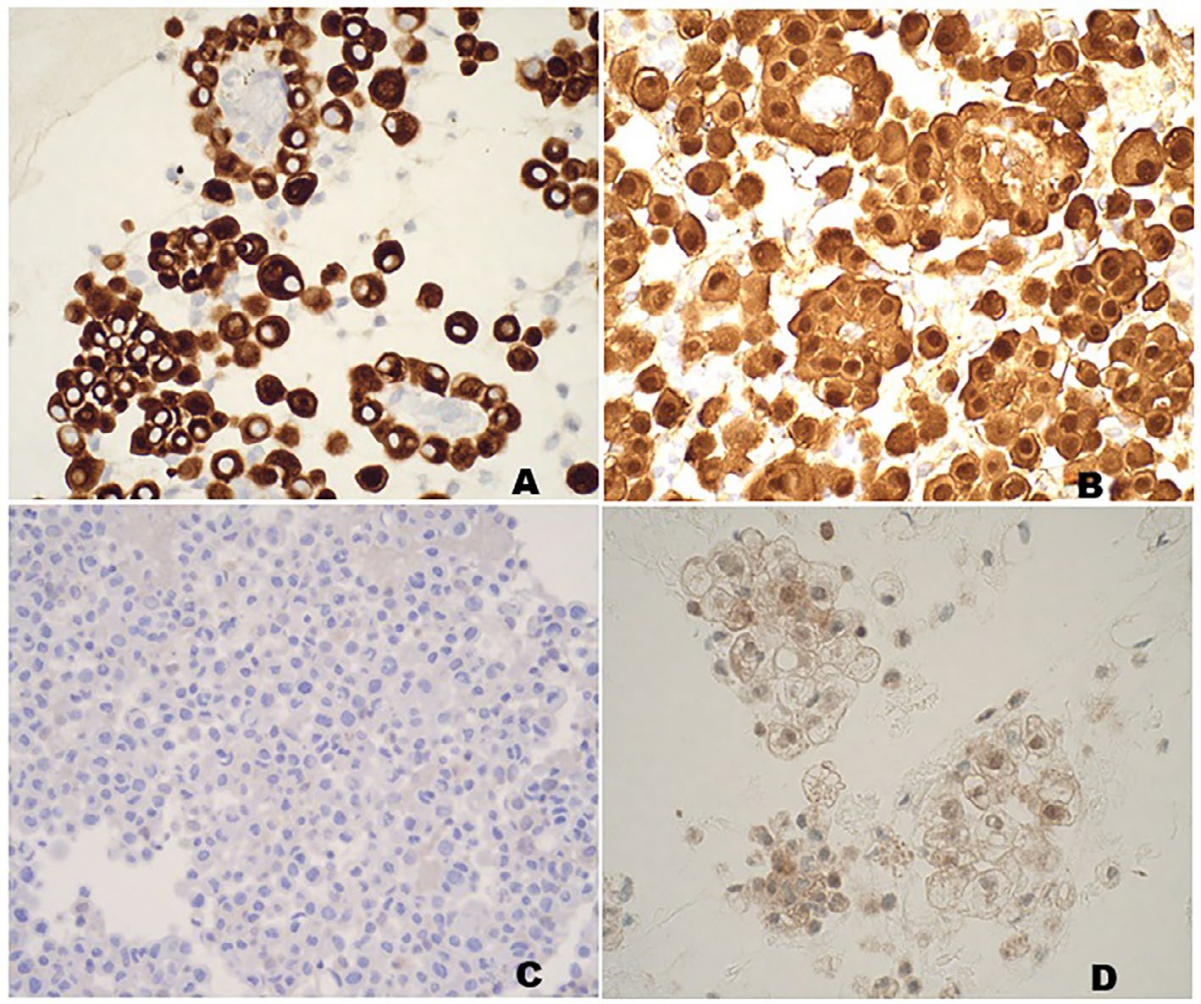
(A) Mesothelioma. Positive cytoplasmic staining for CK5/6 (cell block, 60×). (B) Mesothelioma. Positive cytoplasmic staining for calretinin (cell block, 60×). (C) Mesothelioma. Loss of BAP1 expression (cell block, 40×). (D) Mesothelioma. Loss of MTAP expression (cell block, 60×). [Color figure can be viewed at wileyonlinelibrary.com]

Cytologically, the fluids were very cellular and contained atypical, enlarged mesothelial cells arranged singly (Figure 1a,b) or in combination with cell clusters (Figure 2a,b). The mesothelial cells contained ample cytoplasm resulting in a normal nuclear/cytoplasmic ratio. Noted in the cell block were hollow rings lined by atypical mesothelial cells containing centrally located nuclei with prominent nucleoli. Atypical mesothelial cells, arranged singly and in clusters, were strongly positive for CK 5/6 and calretinin and showed loss of BAP1 and MTAP expression (Figure 3a–d). The WT‐1 nuclear stain was positive in each case. To rule out a diagnosis of adenocarcinoma, claudin 4 and TTF‐1 (SPT24) were performed, each of which were negative in the cases presented. Follow‐up surgical pathology confirmed the diagnosis of mesothelioma in all cases.

## 4 Discussion

The 2024 American Society of Clinical Oncology (ASCO) guidelines for the diagnosis of pleural mesothelioma considers the cytologic diagnosis of pleural fluids only as an initial screening test that should be followed with a biopsy prior to treatment [6]. This recommendation is in contrast to the International System for Reporting Serous Effusions which supports a cytologic diagnosis of mesothelioma in combination with ancillary studies including calretinin, CK 5/6, WT‐1 (mesothelial cell markers and BAP1), MTAP, or FISH for CDKN2A deletion to further delineate benign from malignant mesothelial cells [7, 8].

According to the International Mesothelial Interest Group Guidelines [9], the most valuable biomarkers for distinguishing mesothelioma from benign mesothelial cells include a loss of immunohistochemical expression of BRCA1‐associated protein (BAP1) and the homozygous deletion of the cyclin‐dependent kinase inhibitor 2A (CDKN2A) gene as evaluated by FISH. BAP1 loss is virtually 100% specific in cases of mesothelioma and a sensitivity of 50% to 65% [10]. Methylthioadenosine phosphorylase (MTAP) immunohistochemical loss has emerged as a useful biomarker in the diagnosis of mesothelioma and has shown high specificity for diagnosing mesothelioma with a sensitivity comparable to that of BAP1 and FISH testing [11, 12]. Chapel and colleagues reported high diagnostic specificity and variable sensitivity (0.45–0.65) with MTAP usage, findings similar to FISH analysis [13]. As FISH analysis is costly and labor intensive, some authors recommend BAP1 and MTAP testing as a first line, reserving FISH for the more difficult and challenging cases [12, 13, 14]. FISH analysis was not performed on any of the pleural/peritoneal fluids, as our institution does not perform this test in‐house, leading to time delays in diagnosis. In the current study, three pleural specimens were from patients with a known history of mesothelioma, so the diagnosis was made based on clinical history and the cytologic and IHC findings.

In the current study, prior to 2015, immunohistochemical antibodies to aid in the diagnosis of mesothelioma were limited and FISH analysis was the available option. However, as fresh samples are required for cytogenetic testing which is costly, five effusions were evaluated predominately on cytologic features and diagnosed as suspicious for mesothelioma. With the availability of new biomarkers an accurate diagnosis of mesothelioma was rendered in 18 effusions. Studies [14, 15, 16, 17] have reported that a combination of biomarkers improves the sensitivity in the detection of mesothelioma as opposed to one alone. In a series of 92 cases [15], the sensitivity for BAP1 expression loss was 60.42% and MTAP loss was 43.75%. In combination the sensitivity was 68.8%, with a combined specificity of 84%. Others [16, 17] have reported that an immunopanel of BAP1 and MTAP is 75%–90% sensitive for mesothelioma with high specificity. Due to the limited number of cases in the current study, sensitivity and specificity for biomarker analysis were not calculated; however, in each of the cases, a diagnosis of mesothelioma was made with a high degree of accuracy.

Michael [8] published a recent literature review of the role of cytology in the diagnosis of mesothelioma without surgical intervention. Historically, Warthin [18] in 1897 provided the first rudimentary cytologic description of mesothelioma. It was not until the early 1960s that Naylor [3] and Klempman [19] provided a more detailed and accurate cytologic description but lacked the ancillary testing required to make a definitive diagnosis of mesothelioma. Between the 60s and early 90s, there was controversy as to its diagnostic accuracy; a time period prior to the development of specific markers for mesothelioma and carcinoma. In addition, the diagnosis itself was evolving among surgical pathologists as the histologic criteria for mesothelioma were in development. Over time, the cytologic features were further defined such that the diagnosis of mesothelioma had high specificity and positive predictive value. The advent of biomarker tests including FISH, BAP1, and MTAP provided added value in confirming the diagnosis. In the proper clinical setting and in the hands of experienced cytopathologists/pathologists, the diagnosis of mesothelioma can be made with a high degree of accuracy on cytology. Further evaluation for subtyping and grading are performed on biopsy material.

The cytologic features of mesothelioma on effusions include marked cellularity, cellular aggregates in sheets, morules/clusters or papillary clusters, some containing collagen cores. Morules show knobby boarders. Enlarged, hyperchromatic nuclei contain prominent round nucleoli. As ample cytoplasm is present, the nuclear/cytoplasmic ratio is not increased. Additional features include multinucleation and cellular clasping, as was present in this study. In some cases, an individual cell pattern is present.

An immunohistochemical panel consisting of CK 5/6, calretinin, and WT‐1 are utilized at our institution to establish a mesothelial origin of the cells in the fluid and claudin 4 (epithelial cell marker) are utilized to rule out an adenocarcinoma. TTF‐1 (SPT24) is employed to rule out lung adenocarcinoma, as the majority of effusions with mesothelioma are pleural in nature. BAP1 and MTAP are utilized to further delineate benign from malignant mesothelial cell proliferations.

In conclusion, this study emphasizes the role and the progress cytology has made in combination with immunohistochemistry and biomarkers in the diagnosis of mesothelioma. As pleural/peritoneal effusions are often the first sign of this neoplastic process, discovery at an early stage can lead to more effective treatment and follow‐up management.

## Funding

The author has nothing to report.

## Ethics Statement

The author has nothing to report.

## Consent

The author has nothing to report.

## Conflicts of Interest

The author declares no conflicts of interest.

## Data Availability

Research data are not shared.
